# BioMAJ2Galaxy: automatic update of reference data in Galaxy using BioMAJ

**DOI:** 10.1186/s13742-015-0063-8

**Published:** 2015-05-09

**Authors:** Anthony Bretaudeau, Cyril Monjeaud, Yvan Le Bras, Fabrice Legeai, Olivier Collin

**Affiliations:** 1 INRA, UMR Institut de Génétique, Environnement et Protection des Plantes (IGEPP), BioInformatics Platform for Agroecosystems Arthropods (BIPAA), Campus Beaulieu, Rennes, 35042 France; 2 INRIA, IRISA, GenOuest Core Facility, Campus de Beaulieu, Rennes, 35042 France; 3 INRIA, IRISA, GenScale, Campus de Beaulieu, Rennes, 35042 France

**Keywords:** BioMAJ, Galaxy, Reference data, Data manager, Data libraries

## Abstract

**Background:**

Many bioinformatics tools use reference data, such as genome assemblies or sequence databanks. Galaxy offers multiple ways to give access to this data through its web interface. However, the process of adding new reference data was customarily manual and time consuming, even more so when this data needed to be indexed in a variety of formats (e.g. Blast, Bowtie, BWA, or 2bit).

BioMAJ is a widely used and stable software that is designed to automate the download and transformation of data from various sources. This data can be used directly from the command line, in more complex systems, such as Mobyle, or by using a REST API.

**Findings:**

To ease the process of giving access to reference data in Galaxy, we have developed the BioMAJ2Galaxy module, which enables the gap between BioMAJ and Galaxy to be bridged. With this module, it is now possible to configure BioMAJ to automatically download some reference data, to then convert and/or index it in various formats, and then make this data available in a Galaxy server using data libraries or data managers.

**Conclusions:**

The developments presented in this paper allow us to integrate the reference data in Galaxy in an automatic, reliable, and diskspace-saving way. The code is freely available on the GenOuest GitHub account (https://github.com/genouest/biomaj2galaxy).

## Findings

### Background

#### Reference data in Galaxy

Galaxy [1–3] is a web portal that gives access to a large variety of bioinformatics tools in an easy to use graphical user interface. It also provides visualization modules, such as Trackster [4], which are able to display the analysis results directly in a web browser.

Many of the tools and visualization modules that are present in Galaxy use reference data, such as genomes or specialized databanks (e.g. UniProt [5] or GenBank [6]). This data can be made available in simple flat file formats (e.g. FASTA) or various indexed formats (Bowtie [7], BWA [8], Blast [9]), depending on the requirements of each tool.

There are several methods to configure access to this reference data in Galaxy.

#### *.loc files

The first (and oldest) method available uses so-called ‘*.loc files’. Tabular files with a .loc extension are present in the tool-data directory of the Galaxy server files. Each .loc file contains a list of paths of a given format (e.g. Blast databanks) with a unique identifier and a description.

Although this method is simple and has been working well for a long time, it has a few drawbacks. First, because it is a manual file editing method, administrators have to be very careful to respect the tabular syntax and to give the exact path to the reference files. Furthermore, a restart of the Galaxy server is required for the changes to take effect, which is not very compatible with the high availability of a production server.

#### Data libraries

Reference data can be uploaded to a Galaxy instance in ‘data libraries’. In this case, the data is usually added by administrators of the instance using the Galaxy administration panel. Depending on the configuration, non-administrator users can also be allowed to populate data libraries. Users can then access this data from the ‘data libraries’ menu on the portal and import it to their histories for further analysis.

One important benefit of this method is the ability to restrict access to specific users or user groups, which is very important when adding unpublished data (e.g. newly sequenced genomes).

However, the process of adding data is still manual and as such can be subject to errors. Furthermore, all of the data needs to be imported into a history before it can be used. This implies that Galaxy tools need to be able to use the indexes that are present in a history, which is not the case for many tools at the time of writing this paper.

Using visualizations of a genome stored this way requires each user to create a ‘custom build’, which can be a painful step for users who have little knowledge of the Galaxy interface.

#### Tool data tables and data managers

Instead of reading the list of compatible reference data directly from a unique *.loc file, each tool can load it from a ‘tool data table’. A tool data table is the result of the Galaxy server merging all of the corresponding compatible *.loc files found in its file tree.

‘Data managers’ [10] are a new category of tool that leverage tool data tables, which are available from the main Galaxy Tool Shed [11]. Their goal is to let Galaxy administrators populate tool data tables directly from the administration panel using web forms. Several data managers are already available that allow us to automatically: 1) fetch FASTA files from local or external sites (e.g. NCBI, UCSC); 2) build indexes (e.g. Bowtie, Blast); and, 3) reference this data in the corresponding data tables. They are directly available in the tools that use these data tables.

Tool data tables and data managers are a first move towards the automation of the management of reference data in Galaxy. However, the whole process still requires human intervention to launch the data download and to process it at regular intervals. This method is also Galaxy-centric: the files and indexes that are made available inside Galaxy are stored in the Galaxy server files, following a logical directory structure. Exploring this reference data from outside Galaxy (e.g. with the command line) can be problematic without prior knowledge of this directory structure and of its root. Thus, on architectures where Galaxy is not the only access point to computing resources, this method can lead to data duplication.

#### BioMAJ

BioMAJ [12] is a workflow engine that is dedicated to data synchronization and processing. It automatically finds new releases of reference data on external sources using different protocols (e.g. http, ftp, or sftp). The data is then downloaded and optionally converted to various formats using fully configurable ‘post-processes’.

BioMAJ makes it possible to schedule databank updates automatically at regular intervals, reducing human intervention to a short initial configuration step. Update reports are sent by email if there are unexpected errors.

It is possible to configure BioMAJ and to consult the databank’s status (e.g. latest release, next planned update) using a web user interface. A RESTful API is also available to ease the development of external software requiring access to the databanks.

BioMAJ is used by multiple laboratories and platforms in France and in other countries. A new version is currently under development that will bring many performance improvements and new features while preserving compatibility with existing configuration files.

### BioMAJ2Galaxy presentation

The aim of our project was to use BioMAJ to manage the retrieval and processing of the reference data. We also aimed to extend BioMAJ with a module to manage the reference data that is available in Galaxy.

BioMAJ2Galaxy offers the ability to add or to remove reference data to/from a Galaxy instance. It should be noted that the removal feature can impair the ability of the users of Galaxy to reproduce analyses. However, this feature was developed because it is often impossible to store multiple releases of each databank due to disk space considerations. BioMAJ can be configured to keep a variable number of releases for each databank. At the end of each successful databank update, if this maximum number is reached, then the oldest release is deleted from the disk. Thus, any reference to it in Galaxy also needs to be removed. It is up to the BioMAJ administrators to decide on the number of releases to keep.

**Figure 1:**
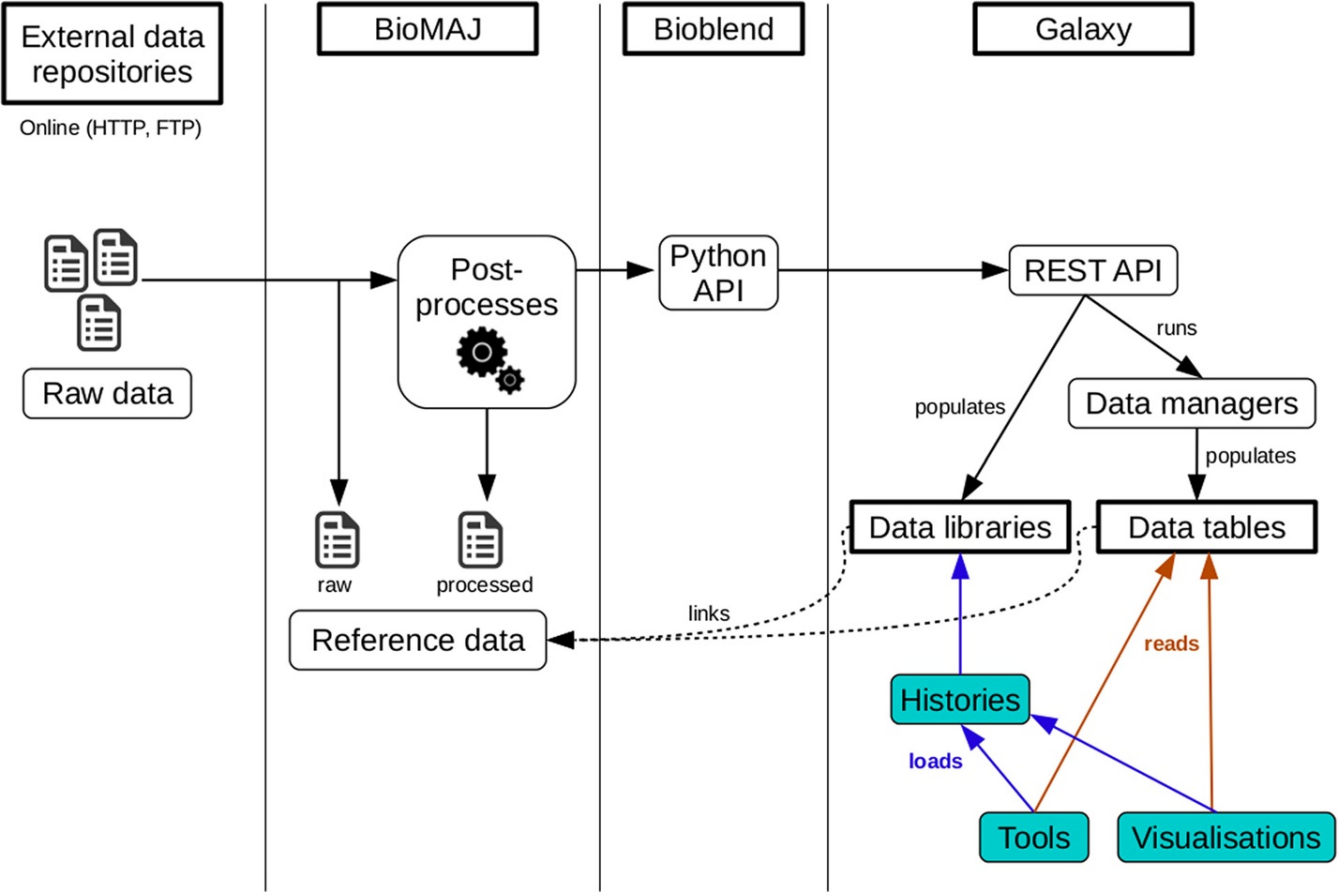
BioMAJ2Galaxy architecture: BioMAJ fetches data from external online repositories. Post-processes are in charge of formatting the data in various formats. They then invoke Galaxy data managers using the Galaxy API via the BioBlend Python library. Alternatively, it is possible to invoke a post-process that adds reference data to the Galaxy data libraries. The injected reference data can then be used directly in tools or visualizations, and/or can be accessed in data libraries. When an obsolete databank version is removed, BioMAJ remove-processes are launched to remove any reference to the corresponding reference data in the Galaxy data tables or data libraries.

BioMAJ2Galaxy takes the form of several Galaxy data managers, BioMAJ post-processes written in Python, and code contributions that were integrated in the Galaxy and BioBlend [13] projects. The code and documentation is freely available on the dedicated GitHub repository [14]. Figure 1 presents a detailed view of the architecture of BioMAJ2Galaxy.

### Data managers

Seven data managers were written for this project, one for each of the most used types of reference data (FASTA, Bowtie, Bowtie2, BWA, 2bit, BlastDB as well as dbkeys). They are all available on the Galaxy User Group Grand Ouest (GUGGO) Tool Shed [15, 16], in the ‘data manager’ category. As such, they can be installed on any recent Galaxy instance directly from the administration panel. These data managers are only in charge of filling the data tables with paths to pre-existing files; they perform no data processing at all. New data managers that are adapted to other formats will be made available on the same Tool Shed in the future, depending on the adoption of the data tables system by new Galaxy tools.

### BioMAJ processes

The data managers presented in the previous paragraph are invoked by Python scripts that are available on the GenOuest GitHub account [14]. This code extensively uses the BioBlend Python library [13] to interact with the Galaxy API.

BioMAJ2Galaxy is composed of four scripts that are used as BioMAJ post-processes (to add new reference data) or remove-processes (to remove obsolete reference data). Because they are standard Python executables, these scripts can also be run manually from the command line.

First, we add the reference data to the corresponding data tables using add_galaxy_data_manager.py. It is possible to add multiple data types at once. A second script, remove_galaxy_data_manager.py, performs the deletion of the reference data from the data tables.

For users who prefer to use data libraries (particularly to restrict access to specific users), add_galaxy_library.py can be used as a post-process to add reference data to a new or already existing data library. An option is available to specify which Galaxy role(s) is allowed to access this data. The script remove_galaxy_data_library.py can be used as a remove-process to delete the reference data from a data library.

These scripts should be launched as the last steps of the processing workflows that are defined for each BioMAJ databank. The previous steps correspond to the data processing tasks that are performed with other post-processes (either BioMAJ built-in scripts or custom scripts).

### Contributions

To have a fully functional system, two modifications in the code of Galaxy were made and then contributed to the official code repository. The first modification [17] adds the ability to remove an item from a data table. Thanks to this modification, BioMAJ is able to remove obsolete reference data from Galaxy (i.e. an old version is replaced by a newer version).

The second modification [18] enables Galaxy to read the content of data tables when creating a new visualization or uploading a new file to a history. This feature was not made available in Galaxy owing to the beta status of data table management in Galaxy.

BioMAJ2Galaxy uses the BioBlend Python library to interact with the Galaxy API. Some changes to this library were needed to give access to new or missing API features (e.g. viewing and editing data tables, data library folder management, and role management). This code has been contributed to the official repository [19] and will be made available in the next stable version of the library.

### Usage

BioMAJ2Galaxy requires an up-to-date version of Galaxy and the data managers from the GUGGO Tool Shed [11] should be installed. An up-to-date version of the BioBlend Python library is also required.

**Figure 2:**
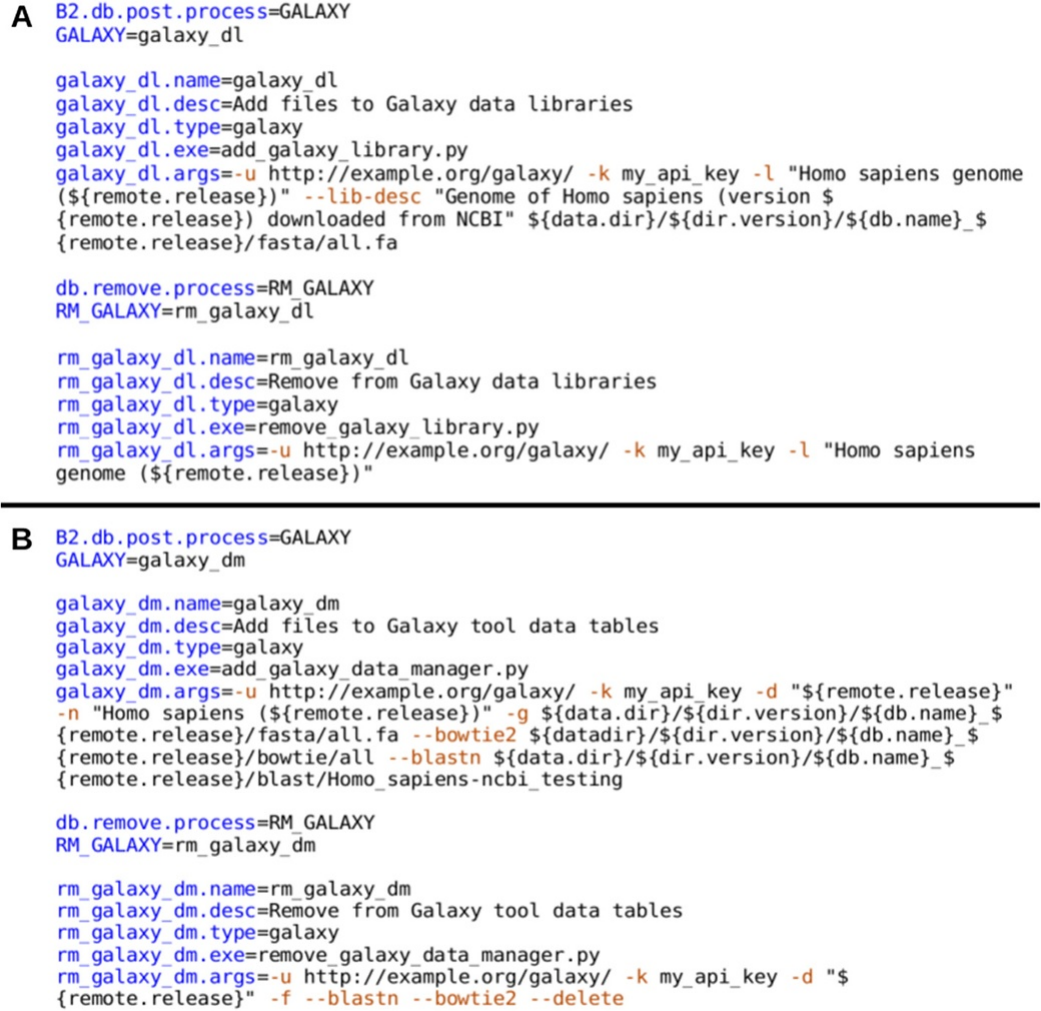
BioMAJ databank configuration examples from a databank configuration file, only the configuration specific to BioMAJ2Galaxy is shown here. More complete example files are available in the BioMAJ archive. **A**: Post-process and remove-process for a databank populating Galaxy data libraries. **B**: Post-process and remove-process for a databank populating Galaxy data tables.

Using BioMAJ2Galaxy for a given BioMAJ databank implies some configuration changes. This can either be done directly from the web interface or by modifying the databank configuration file manually (Figure 2).

Once the configuration step is finished, the databank simply needs to be updated (directly from the web interface, via the command-line, or as a scheduled task). The reference data is available in the whole Galaxy portal as soon as the BioMAJ update process completes with no error.

### Conclusions

We have described BioMAJ2Galaxy, which automates the management of reference data in Galaxy using BioMAJ. One of the main advantages offered by this solution is the ability to automate a time-consuming task. Because human intervention is reduced to a minimum, this system is more reliable than the mostly manual and error-prone tasks that were previously required. Finally, disk space usage is kept as low as possible because each item of reference data is downloaded and formatted only once for all of the access points to the computing resources (e.g. Galaxy [1–3], Mobyle [20], command line, or web services).

BioMAJ2Galaxy is currently being deployed on two production Galaxy instances (GenOuest [21] and BIPAA [22]). Feedback and comments from other teams willing to adopt it are warmly welcomed, either by contacting the authors directly or via the GenOuest platform (support@genouest.org).

Several longer term evolutions are considered, for example by adding the ability to manage access permissions on data tables to Galaxy. The newest developments in BioMAJ should also ease the configuration step in the future by being able to use a redesigned RESTful API.

## Availability and requirements

**Project name:**

BioMAJ2Galaxy

**Project home page:**

https://github.com/genouest/biomaj2galaxy

**Operating system(s):**

Unix-based operating systems

**Programming language:**

Python

**Other requirements:**

BioMAJ, BioBlend library, Galaxy server

**License:**

CeCILL

**Any restrictions to use by non-academics:**

None

## Availability of supporting data

A snapshot of the version of the BioMAJ2Galaxy code used in this paper is archived in the *GigaScience* database GigaDB [23].

## References

[1] Goecks J, Nekrutenko A, Taylor J, the Galaxy Team (2010). Galaxy: a comprehensive approach for supporting accessible, reproducible, and transparent computational research in the life sciences. Genome Biol.

[2] Blankenberg D, Kuster GV, Coraor N, Ananda G, Lazarus R, Mangan M (2010). Galaxy: a web-based genome analysis tool for experimentalists. Curr Protoc Mol Biol.

[3] Giardine B, Riemer C, Hardison RC, Burhans R, Elnitski L, Shah P (2005). Galaxy: a platform for interactive large-scale genome analysis. Genome Res.

[4] Goecks J, Eberhard C, Too T, Nekrutenko A, Taylor J, the Galaxy Team (2013). Web-based visual analysis for high-throughput genomics. BMC Genomics.

[5] The UniProt Consortium (2008). The universal protein resource (UniProt). Nucl Acids Res.

[6] Benson DA, Cavanaugh M, Clark K, Karsch-Mizrachi I, Lipman DJ, Ostell J (2013). Genbank. Nucl Acids Res.

[7] Langmead B, Salzberg SL (2012). Fast gapped-read alignment with Bowtie 2. Nat Methods.

[8] Li H, Durbin R (2009). Fast and accurate short read alignment with Burrows-Wheeler transform. Bioinformatics.

[9] Camacho C, Coulouris G, Avagyan V, Ma N, Papadopoulos J, Bealer K (2009). Blast+: architecture and applications. BMC Bioinform.

[10] Blankenberg D, Johnson JE, Taylor J, Nekrutenko A, The Galaxy Team (2014). Wrangling Galaxy’s reference data. Bioinformatics.

[11] Pennsylvania State University. Galaxy Main Tool Shed. Accessed 04 17 2015. https://toolshed.g2.bx.psu.edu/.

[12] Filangi O, Beausse Y, Assi A, Legrand L, Larré J-M, Martin V (2008). BioMaj: a flexible framework for databanks synchronization and processing. Bioinformatics.

[13] Sloggett C, Goonasekera N, Afgan E (2013). BioBlend: automating pipeline analyses within Galaxy and Cloudman. Bioinformatics.

[14] Genouest BioInformatics Platform. BioMAJ2Galaxy GitHub Repository. Accessed 04 17 2015. https://github.com/genouest/biomaj2galaxy.

[15] GenOuest BioInformatics Platform. GUGGO Tool Shed. Accessed 04 17 2015. http://toolshed.genouest.org.

[16] Biogenouest. GUGGO Web Site. Accessed 04 17 2015. https://www.e-biogenouest.org/groups/guggo.

[17] Galaxy. Galaxy Contribution #577. Accessed 04 17 2015. https://bitbucket.org/galaxy/galaxy-central/pull-request/577/.

[18] Galaxy. Galaxy Contribution #601. Accessed 04 17 2015. https://bitbucket.org/galaxy/galaxy-central/pull-request/601/.

[19] Galaxy Project. BioBlend Contribution #105. Accessed 04 17 2015. https://github.com/afgane/bioblend/pull/105.

[20] Néron B, Ménager H, Maufrais C, Joly N, Maupetit J, Letort S (2009). Mobyle: a new full web bioinformatics framework. Bioinformatics.

[21] GenOuest BioInformatics Platform. GenOuest Galaxy Instance. Accessed 04 17 2015. http://galaxy.genouest.org.

[22] GenOuest BioInformatics Platform. BIPAA Galaxy Instance. Accessed 04 17 2015. http://bipaa-galaxy.genouest.org.

[23] Bretaudeau A, Monjeaud C, Le Bras Y, Legeai F, Collin O. Software and supporting material for “BioMAJ2Galaxy: automatic update of reference data in Galaxy using BioMAJ”. GigaScience Database. 2015. http://doi.org/10.5524/100138. 10.1186/s13742-015-0063-8 PMC4425870 25960870

